# Dietary patterns and associated cardiometabolic risk factors among teachers in Makhado Municipality, South Africa

**DOI:** 10.4102/phcfm.v18i1.5292

**Published:** 2026-08-14

**Authors:** Zelda L. Ratshilivha, Takalani C. Muluvhu, Suzanne Jacobs, Tjale C. Mahopo

**Affiliations:** 1 Department of Biokinetics, Recreation and Sport Science, Faculty of Health Science, University of Venda, Thohoyandou, South Africa; 2 Department of Sport, Rehabilitation and Dental Sciences, Faculty of Science, Tshwane University of Technology, Pretoria, South Africa; 3 Department of Nutrition, Faculty of Health Sciences, University of Venda, Thohoyandou, South Africa

**Keywords:** cardiometabolic diseases, dietary patterns, factor analysis, nutrition transition, teachers, South Africa

## Abstract

**Background:**

Cardiometabolic diseases such as obesity, hypertension, dyslipidaemia and type 2 diabetes are major contributors to morbidity and mortality. Diet is a modifiable risk factor, yet limited evidence exists on its influence on cardiometabolic outcomes among South African teachers.

**Aim:**

To identify dietary patterns and examine their associations with cardiometabolic risk factors among teachers in Makhado Municipality, Limpopo province.

**Setting:**

Rural public schools within Makhado Municipality, Limpopo province, South Africa.

**Methods:**

A cross-sectional study was conducted among 201 teachers selected using stratified random sampling. Data included demographic information, blood pressure, fasting glucose (FG), lipid profiles and anthropometry. Dietary intake was assessed using a validated food-frequency questionnaire that reflected habitual intake. Exploratory factor analysis identified dietary patterns. Associations were analysed using nonparametric tests in STATA version 17 (*p* < 0.05).

**Results:**

Six dietary patterns emerged: mixed, Western, meat-free, Mediterranean fish-free, animal starch product and low-carbohydrates and Mediterranean. The mixed pattern was associated with systolic blood pressure (*p* = 0.03). The Western pattern was associated with total cholesterol (*p* = 0.04) and low-density lipoprotein cholesterol (*p* = 0.02). Meat-free and Mediterranean fish-free patterns were associated with high-density lipoprotein cholesterol (*p* = 0.03), while the animal protein fruit pattern was associated with FG (*p* = 0.02).

**Conclusion:**

Distinct dietary patterns were associated with cardiometabolic risk among teachers, highlighting the need for culturally appropriate dietary interventions.

**Contribution:**

This study provides context-specific evidence that may inform the development of culturally appropriate nutrition interventions, workplace wellness programmes, and primary health care policies aimed at improving cardiometabolic health and reducing the burden of non-communicable diseases among teachers.

## Introduction

Cardiometabolic diseases (CMDs) such as obesity, hypertension, dyslipidaemia and type 2 diabetes remain among the top causes of morbidity and mortality globally, with nutrition recognised as a key modifiable risk factor.^1^ Dietary patterns high in saturated and trans fats, refined carbohydrates, added sugars and salts are strongly associated with increased cardiometabolic risk, whereas diets rich in fruits, vegetables, fibre and healthy fats confer protective effects.^2,3^ For example, an umbrella review found that low-carbohydrate diets, as well as diets like Dietary Approaches to Stop Hypertension (DASH), Nordic and Mediterranean, are associated with significant reductions in body weight, systolic blood pressure (SBP), triglycerides and insulin among adults with at least one cardiometabolic risk factor.^4^ In individuals with type 2 diabetes, different healthy dietary patterns (including plant-based, Mediterranean-style, low-glycaemic index and low-carbohydrate diets) outperform usual diets for improving cardiovascular risk factors, glycated haemoglobin (HbA1c) and body weight.^5^ A recently published systematic review and meta-analysis of randomised controlled trials combining a Mediterranean-type diet with physical activity in high-risk older adults reported significant reductions in waist circumference, visceral fat and triglyceride levels, highlighting the benefits of multicomponent lifestyle interventions.^6^ Sub-Saharan Africa is undergoing a rapid nutrition transition, moving away from traditional diets towards more energy-dense, processed-food patterns, which is contributing to rising rates of overweight, obesity and CMDs.^7^ The implementation of global dietary guidelines in many African contexts is complicated by deeply rooted cultural dietary practices that shape both food choices and meal structures. Across much of sub-Saharan Africa, diets are typically organised around a dominant staple, such as maize, cassava or sorghum, with a smaller portion of relish containing vegetables, legumes or, occasionally, animal-source foods.^8^ While this pattern often provides adequate macronutrient intake, particularly carbohydrates for energy, it can limit dietary diversity and reduce the intake of essential micronutrients. Cultural norms further reinforce these patterns; for instance, meat is frequently reserved for special occasions or used sparingly for flavouring rather than consumed as a primary protein source.^9^ In addition, communal eating practices and the symbolic importance of staple foods influence portion distribution and perceptions of satiety, making individualised dietary recommendations difficult to implement. These cultural factors are closely intertwined with socioeconomic inequalities and food insecurity, which constrain access to a wider variety of nutrient-dense foods. As a result, global dietary guidelines, often developed in high-income settings and based on assumptions about food availability, individual portion control and dietary diversity, may not be directly applicable without contextual adaptation.^10^

Teachers represent an important population group for health promotion interventions, given their influence as role models within school and community settings. However, characterising teachers as uniformly sedentary or as having poor dietary habits may be misleading and context-dependent. While some studies report that educators experience prolonged periods of standing and movement during classroom instruction, others indicate that teaching also involves substantial sedentary tasks such as lesson planning, grading and administrative work.^11,12^ Additionally, occupational stress, time constraints and workplace environments have been associated with suboptimal health behaviours, including irregular meal patterns and low physical activity levels in certain teacher populations.^13^ Evidence from cross-sectional studies in diverse settings suggests that some teachers may be at increased risk of overweight, obesity and related non-communicable diseases, although findings are not consistent across all contexts.^14^ This study examined dietary patterns derived from factor analysis and their associations with cardiometabolic risk factors among schoolteachers in rural South Africa. The results are expected to guide the design of workplace-focused health interventions and contribute to public health policy initiatives aimed at reducing the growing burden of CMDs and inadequate nutrition in resource-constrained settings.

## Research methods and design

### Study design

The study employed a descriptive observational cross-sectional design. Quantitative data were collected at one point in time to describe dietary patterns and cardiometabolic risk factors among teachers and to assess associations between dietary pattern scores and selected cardiometabolic risk categories. The design was appropriate for estimating the burden of cardiometabolic risk factors and exploring associations, but it does not permit causal inference.

### Setting

The study was conducted in rural public schools within Makhado Municipality, located in the Vhembe District of Limpopo province, South Africa. Makhado is one of the largest municipalities in the district and includes a mixture of rural villages, small towns and peri-urban settlements. The area is served mainly by public-sector schools and health services. Many communities in the municipality are affected by socioeconomic constraints, transport limitations and variable access to diverse foods, which may influence dietary practices and cardiometabolic health. The school environment was selected because teachers spend a substantial part of their working day at school and represent a feasible occupational group for workplace health promotion.

### Population and sampling

The target population comprised teachers employed in public schools in Makhado Municipality. The sampling frame consisted of 297 teachers obtained from the Department of Education. Eligible participants were full-time teachers employed at the selected schools who were available during the data collection period and who provided written informed consent. Teachers who were absent during data collection, declined participation, were pregnant or were unable to complete the required measurements were excluded.

The sample size was calculated using Slovin’s formula with a 4% margin of error, which produced a minimum sample size of 201 participants. Stratified random sampling was used to promote representation across the selected schools. The number of teachers selected from each stratum was proportionate to the number of eligible teachers in that stratum. Within each stratum, participants were selected randomly from the staff list until the required sample was reached.

### Data collection procedures

Data were collected by trained research personnel using minimised procedures. Participants completed demographic and dietary questionnaires, after which anthropometric, blood pressure and biochemical measurements were taken. Data collection was scheduled at schools at times agreed with school management to minimise disruption to teaching activities.

### Blood pressure

Blood pressure was measured using an automated sphygmomanometer (Omron Healthcare Inc., United States [US]) after participants had rested in a seated position for at least 5 min. Two readings were obtained, and the average was used for analysis. Blood pressure categories were defined according to the American College of Sports Medicine guidelines: normal (< 120/80 mmHg), prehypertension (120 mmHg – 139/80–89 mmHg), stage 1 hypertension (140/90 mmHg – 159/99 mmHg) and stage 2 hypertension (≥ 160/100 mmHg).^9^

### Cholesterol and glucose screening

Fasting total cholesterol (TC), triglycerides, low-density lipoprotein cholesterol (LDL-C), high-density lipoprotein cholesterol (HDL-C) and glucose levels were measured after a 10-h fast using capillary blood obtained by finger prick. Samples were applied to Polymer Technology Systems (PTS) Panel glucose and lipid test strips and analysed using a CardioChek Plus PA Analyser (Polymer Technology Systems, Inc., US). The analyser was calculated regularly according to the manufacturer’s instructions.

### Anthropometric measurements

Standing height was measured to the nearest 0.1 cm using a stadiometer, with participants barefoot and positioned according to standard procedures. Body mass was measured to the nearest 0.1 kg using a calibrated portable digital scale (seca, Germany). Body mass index (BMI) was calculated as weight in kilograms divided by height in metres squared. Waist circumference was measured to the nearest 0.1 cm using a non-elastic measuring tape in accordance with standard procedures.^9^

### Dietary intake

Habitual dietary intake was assessed using a validated quantitative food-frequency questionnaire (FFQ) adapted to include commonly consumed foods in the study area. Participants reported how frequently they consumed each listed food item during the reference period and provided information on quantities consumed where applicable. Additional questions captured types of cooking oil, added salt use, eating away from home, meal frequency and usual dietary practices. Food items were grouped and used to derive dietary pattern scores.

### Data management and quality assurance

Questionnaires and measurement forms were checked on the day of data collection for completeness and consistency. Data were captured into an electronic database and checked for missing values, implausible entries and out-of-range measurements. A trained research assistant, qualified as a Biokineticist, assisted with measurement procedures and data checking. Any discrepancies were resolved by referring to the original data collection forms. Instrument calibration, repeated measurements and standardised protocols were used to improve reliability.

### Validity and reliability

A trained research assistant, qualified as a Biokineticist, was recruited and oriented in standardised measurement procedures and testing protocols to ensure validity. All instruments were calibrated before data collection. To enhance reliability, blood pressure and anthropometric assessments were repeated, and test–retest correlation coefficients were calculated to evaluate consistency across measurements. A pilot study was conducted in similar schools within Makhado Municipality not included in the main study. Dietary patterns were assessed using standardised FFQs, and the internal consistency of the questionnaire was evaluated using Cronbach’s alpha.

### Data analysis

Descriptive statistics were used to summarise the data. Normality of continuous variables was assessed using Shapiro–Wilk tests and visual inspection of histograms. For comparisons between two groups (male vs female), we used independent samples *t*-tests when variables were approximately normally distributed, and variances were equal (Levene’s test); where normality or equal-variance assumptions were violated, we used the Mann–Whitney *U* test. For understanding of dietary patterns, that is food groupings, exploratory factor analysis (EFA) was undertaken through the principal component analysis. Factors with eigenvalues 1 or above were retained, while the matrix was rotated using the Varimax orthogonal method. All factors with factor loadings of 0.4 or above were considered in the factor to which they belong. For each food group, depending on the individual consumption of that specific week was added to obtain the sum of scores to indicate how often an individual consumes a specific food group in a week. Composite reliability was undertaken to measure the internal consistency using Cronbach alpha. All the dietary patterns with an alpha of 0.7 or above were considered for analysis. To further understand if there are any differences between the dietary group and the cardio metabolic factors, the Kruskal–Wallis test was utilised. To compare the post-estimation differences, Bonferroni pairwise test was used to establish if the differences lie within or between the groups. Effect sizes are reported as Cohen’s *d* for *t*-tests and eta-squared (η^2^) for Kruskal–Wallis tests. All analyses were conducted in STATA version 17 (StataCorp LLC, College Station, TX, US), and statistical significance was set at *p* < 0.05 (adjusted *p*-values reported for multiple comparisons).

### Ethical considerations

Ethical clearance to conduct this study was obtained from the Tshwane University of Technology Research Ethics Committee (No. REC2020/09/002) and Limpopo Provincial Research Ethics Committee (No. REC-111513-038). Informed written consent forms and information sheets were provided to teachers before data collection. To maintain anonymity, participants were assigned unique identification codes instead of personal identifiers on all data collection forms. No names or identifying details appeared on the data collection forms. Completed data collection forms were stored in a locked cabinet accessible only to the principal investigator.

## Results

### Demographic characteristics

Table 1 presents the demographic characteristics of the 201 participants. More than half of the participants were female (*n* = 112; 55.7%), while 89 (44.3%) were male. Participants ranged in age from 23 years to 64 years, with an average age of 40.1 years. Nearly half of the sample was aged 30–44 years (49.3%), followed by those aged 45–65 years (33.3%) and 24–29 years (17.4%).

**TABLE 1:** Teachers’ gender and age group.

Variable	Frequency (*n*)	Percentage (%)
**Gender**		
Female	112	55.7
Male	89	44.3
**Age group (years)**		
24–29	35	17.4
30–44	99	49.3
45–65	67	33.3

*Source:* Ratshilivha ZL, Muluvhu TC, Jacobs S, Mahopo TC. Dietary patterns and associated cardiometabolic risk factors among teachers in Makhado Municipality, South Africa. Afr J Prm Health Care Fam Med. 2026;18(1), a5292. https://doi.org/10.4102/phcfm.v18i1.5292

### Cardiometabolic risk profile by gender

Table 2 summarises cardiometabolic risk factors by gender. There were no significant differences in age between male and female participants (*p* = 0.288). However, significant gender differences were observed in several anthropometric measures. Female teachers had significantly higher body weight (*p* < 0.001), BMI (*p* < 0.001) and waist circumference (*p* < 0.001) compared to their male counterparts, indicating a greater burden of adiposity among women. In contrast, males were significantly taller than females (*p* = 0.002). With regard to biochemical markers, female teachers exhibited significantly higher TC (*p* = 0.006) and triglyceride levels (*p* = 0.005), suggesting a less favourable lipid profile. No significant gender differences were found for systolic or diastolic blood pressure, high-density lipoprotein (HDL) cholesterol, low-density lipoprotein (LDL) cholesterol or fasting glucose (FG) levels (*p* > 0.05). Overall, these findings indicate that female teachers in this sample may be at greater risk for cardiometabolic disorders, particularly those associated with obesity and dyslipidaemia.

**TABLE 2:** Cardiometabolic risk factors for teachers by gender.

Variable	Total group (Mean ± s.d.)	Female (Mean ± s.d.)	Male (Mean ± s.d.)	*p*-value
Age (years)	40.1 ± 10.3	40.8 ± 10.9	39.2 ± 9.5	0.288
Height (cm)	1.6 ± 0.0	1.6 ± 0.0	1.7 ± 0.0	0.002*
Weight (kg)	81.0 ± 19.4	84.5 ± 18.7	76.5 ± 19.6	0.000*
Body mass index (kg/m^2^)	29.9 ± 7.5	32.8 ± 7.3	26.2 ± 6.0	0.000*
Waist circumference (cm)	94.3 ± 14.4	97.7 ± 15.2	90.0 ± 12.1	0.000*
Systolic blood pressure (mmHg)	132.2 ± 20.3	130.3 ± 22.3	134.7 ± 16.9	0.065
Diastolic blood pressure (mmHg)	82.0 ± 13.6	81.3 ± 14.0	82.9 ± 13.2	0.212
Total cholesterol (mmol/L)	4.5 ± 1.5	4.8 ± 1.6	4.2 ± 1.2	0.006*
Triglyceride (mmol/L)	1.9 ± 1.2	2.1 ± 1.2	1.6 ± 1.1	0.005*
High-density lipoprotein (mmol/L)	1.37 ± 0.48	1.35 ± 0.57	1.38 ± 0.35	0.335
Low-density lipoprotein (mmol/L)	3.04 ± 1.68	2.96 ± 1.30	3.14 ± 2.07	0.225
Fasting glucose (mmol/L)	6.62 ± 2.57	6.70 ± 2.62	6.53 ± 2.52	0.326

*Source:* Ratshilivha ZL, Muluvhu TC, Jacobs S, Mahopo TC. Dietary patterns and associated cardiometabolic risk factors among teachers in Makhado Municipality, South Africa. Afr J Prm Health Care Fam Med. 2026;18(1), a5292. https://doi.org/10.4102/phcfm.v18i1.5292

s.d., standard deviation.

* , Significant at 0.05.

### Dietary patterns identified by exploratory factor analysis

Table 3 presents the EFA results identifying distinct dietary patterns among teachers, along with measures of internal consistency and item correlations. Seven dietary patterns were extracted, each defined by food items with factor loadings greater than 0.40, indicating meaningful contributions to the respective patterns.

**TABLE 3:** Factor analysis and correlation of dietary patterns among teachers.

Dietary pattern/food item	Factor loadings	Item–Rest correlation	Cronbach’s alpha if item deleted	Overall α	Proportion for factor
**Mixed diet**	-	-	-	0.8759	0.0900
Oats	0.5129	0.4838	0.8706	-	-
All bran	0.6742	0.5804	0.8668	-	-
Boiled eggs	0.7391	0.6576	0.8623	-	-
**Starch animal product**	-	-	-	0.8068	0.0700
Mabele	0.4115	0.4133	0.3488	-	-
Fried beef	0.7267	0.7095	0.6197	-	-
**Western diet**	-	-	-	0.8050	0.0536
Morvite	0.6965	0.7593	0.7186	-	-
Plain yoghurt	0.8029	0.9283	0.8430	-	-
**Meat-free diet**	-	-	-	0.7200	0.0447
Boiled green cabbage	0.4686	0.6372	0.5256	-	-
Boiled fresh thanga	0.6518	0.7111	0.5619	-	-
**Low carbohydrate/Mediterranean diet**	-	-	-	0.7281	0.0394
Boiled chicken	0.6470	0.8649	0.6724	-	-
Salad carrots	0.5631	0.4392	0.3536	-	-
**Mediterranean fish-free diet**	-	-	-	0.6689	0.0348
Squash	0.6749	0.8022	0.5806	-	-
100% fruit juice	0.6921	0.8339	0.5894	-	-
**Animal protein fruit diet**	-	-	-	0.7538	0.0318
Boiled canned fish	0.8510	0.7875	0.7185	-	-
Medium orange	0.7942	0.7962	0.6320	-	-

Note: α = Cronbach’s alpha coefficient, indicating internal consistency reliability. Factor loadings > 0.40 were considered significant. Proportion for factor represents the percentage of total variance explained by each extracted factor.

The *Mixed Diet pattern* demonstrated high internal consistency (Cronbach’s α = 0.8759) and included oats, all bran and boiled eggs, all with moderate-to-strong factor loadings (0.51–0.74) and acceptable item–rest correlations. This suggests a relatively balanced dietary pattern incorporating both plant- and animal-based foods. Similarly, the *Western Diet pattern* (α = 0.8050) showed strong internal reliability, with high loadings for Morvite and plain yoghurt, particularly yoghurt, which exhibited a very strong item–rest correlation (0.9283), indicating a strong contribution to this pattern.

The *Starch Animal Product pattern* (α = 0.8068) was characterised by mabele and fried beef, although mabele showed a comparatively lower factor loading (0.41) and weaker internal consistency contribution, suggesting some heterogeneity within this pattern. The *Meat-Free Diet pattern* (α = 0.7200) included boiled green cabbage and fresh thanga, with moderate factor loadings and acceptable correlations, reflecting a plant-based dietary pattern with reasonable internal consistency.

The *Low Carbohydrate* and *Mediterranean Diet pattern* (α = 0.7281) included boiled chicken and salad carrots, indicating a combination of lean protein and vegetables, although variability in item–rest correlations suggests moderate coherence. In contrast, the *Mediterranean Fish-Free Diet pattern* (α = 0.6689) and the *Animal Protein Fruit Diet pattern* (α = 0.7538) showed lower proportions of explained variance and moderate reliability, indicating that these patterns are less dominant within the sample.

Overall, the proportion of variance explained by each factor was relatively low (ranging from 3.18% to 9%), suggesting that dietary behaviours among teachers are diverse and not strongly dominated by a single pattern. Nevertheless, the generally acceptable Cronbach’s alpha coefficients (≥ 0.70 for most patterns) indicate that the identified dietary patterns demonstrate reasonable internal consistency and can be considered reliable representations of habitual dietary intake in this population.

### Dietary patterns and cardiometabolic risk factors

Table 4 shows the differences between dietary pattern scores and cardiometabolic risk factors using nonparametric comparisons, the Kruskal–Wallis. There was no statistically significant difference in mixed diet scores across BMI categories (χ^2^ = 2.79, *p* = 0.42), nor for the starch animal product pattern (χ^2^ = 4.11, *p* = 0.24), indicating that adherence to these dietary patterns did not vary by weight status.

**TABLE 4:** Differences in dietary pattern scores across cardiometabolic risk-factor categories.

Dietary pattern	Risk factor and categories compared	Rank sum/summary from output	*p*	Bonferroni-adjusted post-hoc comparison
Mixed diet	BMI: underweight, normal, overweight, obese	418.5; 5776.5; 5395.5; 8710.5	0.42	Not significant
Mixed diet	SBP: normal, prehypertension, stage 1 hypertension	4749.0	0.03*	Stage 1 hypertension vs prehypertension: mean rank difference = 2.90, *p* < 0.001
Starch-animal product	BMI: underweight, normal, overweight, obese	334.5	0.24	Not significant
Western diet	TC: desirable, borderline, high	13564.5	0.04*	High vs desirable cholesterol: mean rank difference = -2.16, *p* = 0.04
Meat-free pattern	HDL-C: optimal, borderline, at risk	11901.0	0.03*	At risk vs optimal HDL-C: mean rank difference = -2.34, *p* = 0.02
Mediterranean fish-free pattern	HDL-C: optimal, borderline, at risk	1278.5	0.03*	At risk vs optimal: -2.02, *p* = 0.03; at risk vs borderline: -2.29, *p* = 0.03
Animal protein-fruit pattern	FG: normal, prediabetes, diabetes	6666.5	0.02*	Diabetes vs prediabetes: -2.44, *p* = 0.02; diabetes vs prediabetes: -2.24, *p* = 0.03 (verify duplicate comparison)
Animal protein-fruit pattern	HDL-C: optimal, borderline, at risk	12020.0	0.03*	At risk vs optimal: -2.16, *p* = 0.04; at risk vs borderline: 2.16, *p* = 0.04
Animal protein-fruit pattern	SBP: normal, prehypertension, stage 1 hypertension	5797.0	0.01*	Stage 1 hypertension vs normal: 3.14, *p* < 0.001

Note: Kruskal–Wallis tests were used for overall comparisons. Significant differences are marked with an asterisk.

SBP, systolic blood pressure; BMI, body mass index; TC, total cholesterol; HDL-C, high-density lipoprotein cholesterol; FG, fasting glucose.

However, significant differences were observed for severaldietary patterns in relation to specific cardiometabolic variables. Mixed diet scores differed significantly across SBP categories (χ^2^ = 8.70, *p* = 0.03). Post hoc pairwise comparisons indicated that participants with stage 1 hypertension had significantly higher mean ranks compared to those with prehypertension (mean difference = 2.9, *p* < 0.001).

Western diet scores differed significantly across TC categories (χ^2^ = 6.11, *p* = 0.04), with significantly lower scores observed in participants with high TC compared to those with desirable levels (mean difference = −2.16, *p* = 0.04). Similarly, meat-free diet scores varied significantly across HDL categories (χ^2^ = 6.96, *p* = 0.03), with participants classified as ‘at risk’ demonstrating significantly lower scores than those with optimal HDL levels (mean difference = −2.34, *p* = 0.02).

For the Mediterranean fish-free diet pattern, there was a significant difference across HDL categories (χ^2^ = 6.51, *p* = 0.03). Pairwise comparisons showed that participants in the at-risk group had significantly lower scores than those in both the optimal (mean difference = −2.02, *p* = 0.03) and borderline categories (mean difference = −2.29, *p* = 0.03).

The animal protein fruit diet pattern demonstrated significant differences across multiple outcomes. Scores differed across FG categories (χ^2^ = 7.68, *p* = 0.02), with significantly lower scores observed in participants with diabetes compared to those with prediabetes (mean differences = −2.44, *p* = 0.02; −2.24, *p* = 0.03). Significant differences were also observed across HDL categories (χ^2^ = 6.96, *p* = 0.03), where at-risk participants had lower scores compared to optimal and borderline groups. In addition, scores differed significantly across SBP categories (χ^2^ = 10.36, *p* = 0.01), with higher scores observed among participants with stage 1 hypertension compared to those with normal blood pressure (mean difference = 3.14, *p* < 0.001).

Overall, these results indicate that dietary pattern scores were significantly associated with selected cardiometabolic risk factors, particularly lipid profiles, FG and SBP, while no significant associations were observed with BMI.

## Discussion

A combination of common food groups constitutes distinct dietary patterns. In this study, the ‘mixed’ dietary pattern differed significantly across SBP categories, suggesting an association between a mixed diet and SBP, particularly between participants with stage 1 hypertension and prehypertension.^12^ Recent observational studies report similar links between empirically derived ‘mixed’ or non-prudent dietary patterns and elevated blood pressure, supporting the plausibility of the study finding.^13^ These findings contradict those of Wirnitzer et al.,^12^ who reported that most schoolteachers consuming a mixed diet had a higher BMI. In contrast, Holmes et al.^11^ found no association between the ‘mixed diet’ pattern and overweight or obesity.

This study found a statistically significant difference in the ‘Western’ dietary pattern across cholesterol categories, indicating a potential association between Western-style diets and higher TC/LDL-C. This is consistent with prior work showing that Western or processed-food patterns are linked to dyslipidaemia and adverse lipid profiles in diverse populations.^4,5^ Similarly, overweight and obesity increased with greater adherence to Western dietary habits among teachers in Malaysia.^14,15^

The ‘meat-free’ (plant-based/vegetarian) dietary patterns observed in this study were linked to variations in HDL categories. Evidence from prior research shows that vegetarian and vegan diets consistently reduce TC and LDL-C levels, although their impact on HDL cholesterol remains inconsistent and appears to vary according to dietary composition and study methodology.^16,17^

For Mediterranean-type patterns, the *p*-value for BMI (0.09) suggested a trend but not a statistically significant relationship in our sample. However, large, randomised trials and systematic reviews consistently document improvements in lipid function, glycaemic control and some HDL outcomes with Mediterranean dietary interventions, supporting the biological plausibility of beneficial effects despite null BMI findings here.^18,19^ This is in line with Mestre et al.,^15^ who found no significant association between Mediterranean diet adherence and BMI.

The study observed significant differences across LDL categories for the ‘animal starch product’ pattern and significant differences in FG, HDL cholesterol and SBP for other locally derived patterns. Evidence on highly specific, locally defined patterns such as ‘animal starch product’, ‘Mediterranean fish-free’ and region-specific starch-protein combinations is sparse; most large studies group foods into broader Western, prudent or plant-based patterns, so direct comparisons are limited. Some literature suggests that starch quality and the animal compared to plant origin of protein and fats modulate glucose and lipid metabolism, which supports these study findings but highlights the need for more context-specific research.^20,21^

This study’s results broadly align with literature linking processed/Western patterns to adverse lipid profiles and support evidence that plant-forward and Mediterranean patterns influence lipid metabolism and blood pressure. However, the specificity of several novel, locally relevant patterns identified in this study highlights gaps in the literature and the need for longitudinal and intervention research in similar low-resource and rural settings to determine directionality and causality.^16,17,20^

These findings highlight the importance of tailoring workplace-based health interventions to address teachers’ dietary behaviours, given their dual role as both at-risk individuals and community role models. Promoting healthier eating patterns, reducing consumption of Western-style foods and encouraging plant-based and Mediterranean-inspired diets could improve cardiometabolic outcomes in this group. At a policy level, integrating nutrition education and workplace wellness programmes into the education sector may provide a sustainable avenue to address the rising burden of CMDs in resource-limited rural settings.^22^

Overall, this study provides novel evidence on the relationship between dietary patterns and cardiometabolic risk factors among rural South African teachers, highlighting the urgent need for culturally relevant interventions to promote healthier diets and reduce CMD risk in this vulnerable population.

### Limitations

This study should be interpreted considering several limitations. Firstly, the cross-sectional design permits identification of associations but does not establish temporal direction or causality between dietary patterns and cardiometabolic risk factors. Secondly, dietary intake was assessed using an FFQ, which is subject to recall bias, reporting errors and social desirability bias. Thirdly, the dietary patterns were empirically derived from the current sample, and the labels assigned to factors were based on the food items with the highest loadings; therefore, the patterns may not be directly comparable with standard dietary indices used in other studies. Fourthly, some factors included only a small number of food items and explained a relatively low proportion of variance, which may limit interpretability. Fifthly, residual confounding by physical activity, medication use, socioeconomic status, stress, smoking, alcohol use and total energy intake may have influenced the observed associations. Finally, the study was conducted among teachers in one municipality, which may limit generalisability to teachers in other regions or to the general adult population.

### Recommendation

Schools and local health authorities should implement nutrition education programmes targeting teachers to encourage healthier dietary choices, particularly reducing reliance on ‘Western’ diets high in saturated fats and processed foods. Stakeholders in the education and health sectors should collaborate to promote access to healthier meals within school settings, potentially introducing workplace wellness programmes tailored to teachers. Given the limited literature on ‘animal starch product’, ‘meat-free’ and ‘Mediterranean fish-free’ dietary patterns, additional studies are recommended to explore their long-term impact on lipid profiles and cardiometabolic risk. Regular screening of blood pressure, cholesterol and BMI among teachers should be integrated into occupational health programmes to identify and manage early signs of cardiometabolic risk. Teachers, as role models within communities, should be empowered to adopt and promote healthy lifestyle practices, potentially influencing students and families towards healthier dietary behaviours.

## Conclusion

This study identified significant associations between dietary patterns and cardiometabolic risk factors among teachers in the Vhembe District. The ‘mixed’ dietary pattern was linked to differences in SBP, while adherence to a ‘Western’ diet correlated with higher LDL cholesterol levels. Although the ‘low-carbohydrate/Mediterranean’ pattern showed a non-significant trend across BMI categories, the ‘animal starch product’, ‘meat-free’ and ‘Mediterranean fish-free’ patterns were associated with variations in lipid profiles. Overall, these findings underscore the complex influence of diet on cardiometabolic health and highlight the importance of dietary behaviour in managing cardiovascular risk among teachers.
